# Combination Proximal Pulmonary Artery Coiling and Distal Embolization Induces Chronic Elevations in Pulmonary Artery Pressure in Swine

**DOI:** 10.1371/journal.pone.0124526

**Published:** 2015-04-29

**Authors:** Jaume Aguero, Kiyotake Ishikawa, Kenneth M. Fish, Nadjib Hammoudi, Lahouaria Hadri, Ana Garcia-Alvarez, Borja Ibanez, Valentin Fuster, Roger J. Hajjar, Jane A. Leopold

**Affiliations:** 1 Cardiovascular Research Center, Icahn School of Medicine at Mount Sinai, New York, New York, United States of America; 2 Centro Nacional de Investigaciones Cardiovasculares Carlos III (CNIC)- Epidemiology, Atherothrombosis and Imaging Department, Madrid, Spain; 3 Zena and Michael A. Wiener Cardiovascular Institute, Icahn School of Medicine at Mount Sinai, New York, New York, United States of America; 4 Cardiovascular Medicine Division, Brigham and Women’s Hospital, Harvard Medical School, Boston, Massachusetts, United States of America; VU University Medical Center, NETHERLANDS

## Abstract

Pulmonary hypertension (PH) is associated with aberrant vascular remodeling and right ventricular (RV) dysfunction that contribute to early mortality. Large animal models that recapitulate human PH are essential for mechanistic studies and evaluating novel therapies; however, these models are not readily accessible to the field owing to the need for advanced surgical techniques or hypoxia. In this study, we present a novel swine model that develops cardiopulmonary hemodynamics and structural changes characteristic of chronic PH. This percutaneous model was created in swine (n=6) by combining distal embolization of dextran beads with selective coiling of the lobar pulmonary arteries (2 procedures per lung over 4 weeks). As controls, findings from this model were compared with those from a standard weekly distal embolization model (n=6) and sham animals (n=4). Survival with the combined embolization model was 100%. At 8 weeks after the index procedure, combined embolization procedure animals had increased mean pulmonary artery pressure (mPA) and pulmonary vascular resistance (PVR) compared to the controls with no effect on left heart or systemic pressures. RV remodeling and RV dysfunction were also present with a decrease in the RV ejection fraction, increase in the myocardial performance index, impaired longitudinal function, as well as cardiomyocyte hypertrophy, and interstitial fibrosis, which were not present in the controls. Pulmonary vascular remodeling occurred in both embolization models, although only the combination embolization model had a decrease in pulmonary capacitance. Taken together, these cardiopulmonary hemodynamic and structural findings identify the novel combination embolization swine model as a valuable tool for future studies of chronic PH.

## Introduction

Pulmonary hypertension is associated with pathological pulmonary vascular remodeling as well as maladaptive RV remodeling and, ultimately, RV failure that leads to poor clinical outcomes and early death [1]. Vascular remodeling occurs predominantly, but not exclusively, in the pulmonary arterioles and manifests as intimal and medial hypertrophy, inflammation, thrombosis, and fibrosis that disrupts the normal vessel architecture and leads to subtotal luminal obliteration. The RV also undergoes structural and functional remodeling with evidence of hypertrophy and impaired indices of ventricular contraction and relaxation ^1^. The finding of pathologically remodeled vessels and RV dysfunction is shared among WHO pulmonary hypertension (PH) Groups, although the pathogenesis of Group 1 PH originates within the vasculature in contradistinction to other forms of PH (Groups 2–5) where pulmonary arteriole remodeling occurs as a consequence of sustained hemodynamic disturbances [2].

Large animal models that are representative of human PH are necessary to bridge the gap between studies in the well-accepted rodent models of experimental PH and clinical testing [3]. Large animal models also offer a unique opportunity to study PH using typical clinical diagnostic methodologies, such as right heart catheterization and advanced imaging techniques, together with a comprehensive characterization of the disease at a cellular and molecular level. This has led to attempts to develop accessible large animal models that recapitulate the pathological and clinical features of human PH. Several models of PH have been created in large animals using advanced surgical procedures such as the subclavian-pulmonary artery shunt overcirculation and pulmonary vein stenosis models [4,5,6]. To overcome the complexity associated with these surgical models, recurrent pulmonary vascular embolization to obstruct the distal pulmonary arterioles has been utilized as a methodology to create a model of PH (Group 4) [7,8,9,10,11,12,13]; however, studies of this model have reported mixed results with some protocols failing to achieve PH cardiopulmonary hemodynamics. There is also no consensus pertaining to the choice of embolic material, timing of administration, and the number of embolization procedures required to create the model. In addition, many of the studies did not evaluate RV function and only examined hemodynamic changes at a time-point <1 month after the final embolization procedure thereby calling into the question the long-term durability of those models.

Owing to the lack of information about longer-term pulmonary hemodynamics and RV function in a large animal pulmonary embolization model, the aim of the present study was to determine if this model could develop chronic PH with characteristics of human disease or if the model required modification to achieve chronic PH. We, therefore, hypothesized that only a pulmonary embolization protocol that was sufficient to produce pulmonary vascular (sub)total occlusion would create a model of chronic PH with RV dysfunction. To test this hypothesis, we utilized a novel embolization protocol that combined repeated distal embolization with microspheres, similar to prior studies, with proximal pulmonary coil embolization using silk suture fragments and examined cardiopulmonary hemodynamics and structural changes at 8 weeks.

## Methods

### Experimental design

Sixteen female Yorkshire pigs were included in the study. The pigs were housed in the animal facility until the final follow-up assessment. At the end of the study, animals were euthanized with sodium pentobarbital and the heart and lungs were resected for analysis. The study was performed in accordance with the Guidelines for the Care and Use of Laboratory Animals and was approved by the Icahn School of Medicine at Mount Sinai Institutional Animal Care and Use Committee.

### Peri-procedural anesthesia

For procedures related to creating the model and at final follow-up, animals were pre-medicated using Telazol (tiletamine/zolazepam) 6.0 mg/kg, intubated, and ventilated with 40% oxygen at 10 ml/kg tidal volume and 15 respirations per minute to maintain an end-tidal CO_2_ between 35–45 mmHg. General anesthesia was maintained throughout the procedures with propofol 5–8 mg/kg/hr.

### Recurrent embolization model of chronic PH

**Fig 1:**
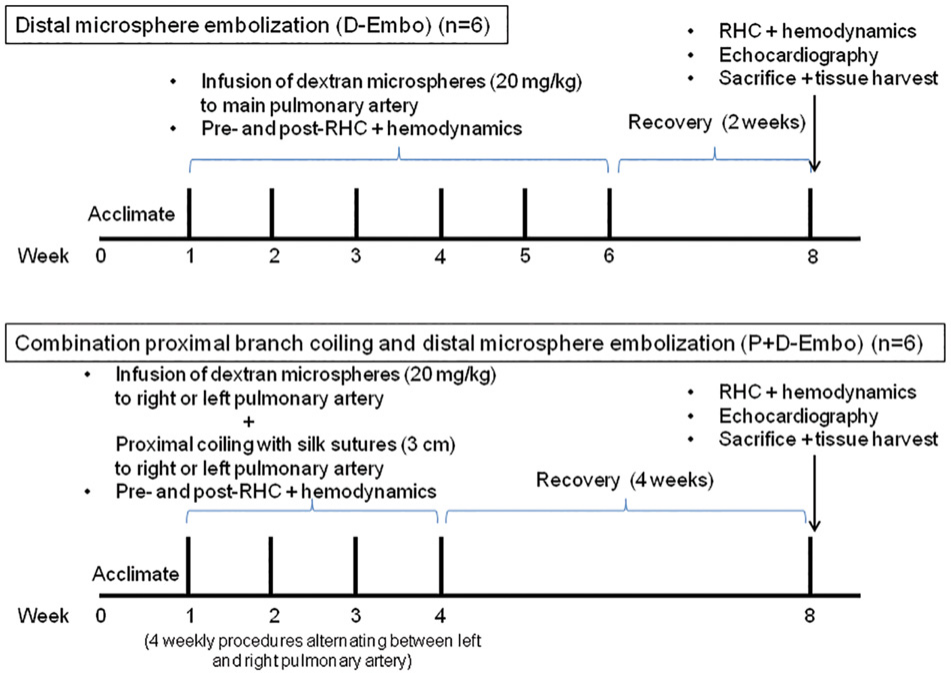
Study design and experimental protocol.

Two different recurrent pulmonary embolization protocols were utilized to compare the standard distal embolization model (D-Embo) with the combination proximal coiling and distal embolization model (P+D-Embo). The final follow-up assessment was planned for week 8, which was at least 2 weeks after the last embolization procedure. A sham group (n = 4) that did not undergo embolization served as a control and was used as a benchmark of changes over time related to somatic growth. The experimental protocol is shown in Fig 1.

#### Distal embolization model (D-Embo)(n = 6)

First, pulmonary angiography was performed using a 5F pigtail catheter to identify branches of the right and left main pulmonary arteries. A 7.5 Fr Swan-Ganz catheter (Edwards Lifesciences) was advanced to the right or left pulmonary artery via femoral vein access and dextran microspheres (100–300 μm diameter, coarse Sephadex G-50, Sigma-Aldrich) at a dose of 20 mg/kg were infused through the lumen of the Swan-Ganz catheter on a weekly basis. The dextran microspheres were mixed with 30 ml of sterile 0.9% sodium chloride at least 6 hours prior to the injection. Using this approach, all animals recovered with 100% survival. This dosing regimen was based on the results of a pilot study that demonstrated that infusion of concentrated microspheres (i.e., without dilution in saline) at doses as low as 10 mg/kg led to severe respiratory distress and high early mortality. Once the microspheres were diluted in saline prior to infusion doses as high as 15–20 mg/kg were tolerated without acute morbidity or mortality.

#### Proximal + distal embolization model (P+D-Embo) (n = 6)

This model combined distal microsphere embolization with coiling of multiple pulmonary artery branches. To avoid acute severe PH and RV failure as a result of acute bilateral obstruction of the pulmonary arteries, the distal embolization procedure was modified. Using a 7.5 Fr Swan-Ganz (Edwards, Lifesciences) catheter, 20 mg/kg of dextran microspheres were injected through the distal port while the balloon was inflated in the right pulmonary artery. The Swan-Ganz catheter was exchanged for a 5F JR 3.5 coronary guide catheter (Cordis) followed by selective coiling of the main branches of the right pulmonary artery. The coiling procedure was performed by cutting sterile silk sutures into 3 cm long sections and selectively deploying these segments into each branch. The following week, the same procedures were performed on the contralateral lung. Over a period of 4 weeks, the embolization procedures were performed 2 times for each lung per animal.

### Hemodynamic assessment

Cardiopulmonary hemodynamics were recorded using a 7.5 Fr Swan-Ganz catheter (Edwards Lifesciences) that was advanced to the heart via femoral vein access to measure mean right atrial (RA) pressure, mean pulmonary artery pressure (mPA), pulmonary artery occlusion pressure (PAOP), and cardiac output (CO) was measured using the thermodilution method. Pulmonary vascular resistance (PVR) was calculated as (mPAP-PAOP)/CO, and pulmonary artery capacitance as SV/PP, where SV is stroke volume and PP is pulse pressure. CO, SV, PVR and pulmonary artery capacitance were indexed to the body surface area as previously reported [14]. All parameters were recorded during brief periods of end-expiratory breath-hold in anesthetized and ventilated animals.

### Echocardiography

Echocardiographic data were collected at baseline and final follow-up in all animals using a Philips iE33 ultrasound system (Philips Medical Systems, Andover, MA, USA). Cardiac performance was analyzed by measuring the following parameters: 1) anatomical M-mode tricuspid annulus plane systolic excursion (TAPSE); 2) tissue Doppler-derived tricuspid annular systolic velocity; 3) myocardial performance index (MPI); and 4) RV end-systolic and end-diastolic volumes and RV ejection fraction (RVEF) that were obtained from 3D datasets that were analyzed offline with QLAB software (Philips Medical Systems, Andover, MA, USA). All parameters were averaged from three consecutive measurements. For RV volumetric data, interobserver variability was assessed in a subset of 10 animals and estimated using the intraclass correlation coefficient. This parameter was 0.81, 0.96 and 0.93 for end-diastolic volume, end-systolic volume and RVEF, respectively.

### Heart and lung morphology

To assess relative RV hypertrophy, the heart was sectioned into RV and left ventricle (LV), weighed, and RV hypertrophy was assessed using the Fulton index (RV/LV + septum). After harvests, the heart and lung tissue samples were placed in 10% formaldehyde solution, fixed, processed and tissue blocks were embedded in paraffin. From randomly selected segments harvested from the upper and lower lobes from both lungs, the presence and type of vascular lesions were assessed from 5 μm thick sections stained with Masson's trichrome or hematoxylin and eosin. Distal pulmonary artery remodeling was assessed in 10–12 randomly identified vessels and was quantified by the relative medial thickness (MT) measured as: MT = (WTx2)x100/ED (WT = wall thickness and ED = external diameter) at different vessel diameters. Cardiomyocyte cross-sectional area (CSA) was measured in cardiac sections stained with wheat germ agglutinin (WGA) (conjugated to Oregon Green 488, 10 μg/mL, Invitrogen) and co-stained with phalloidin (conjugated to Alexa fluor 546, 165 nM, Invitrogen). Images of RV cardiomyocyte cell membranes were captured digitally and analyzed using ImageJ software (National Institutes of Health). RV myocardial fibrosis was assessed with Masson’s Trichrome staining, quantified, and reported as % area.

### Statistical analysis

All continuous variables are expressed as the mean ± SD. The distribution of continuous variables was assessed graphically using histograms. Normality was determined using Q-Q plots Continuous variables were compared between three groups using one-way analysis of variance or the non-parametric Kruskal-Wallis test, followed by *post hoc* analysis (Tukey HSD method to correct for multiple pairwise comparisons). As the main objective of the study was the development of chronic PVD, hemodynamic changes were computed as the difference between the last follow-up (8 weeks) and the baseline values. For quantitative analyses of histological samples, a mixed model regression was used considering the Group as a fixed factor and the animals within groups as random factors to account for nested measurements within each animal. All statistical analyses were performed using R software version 3.1.0 (http://cran.r-project.org/).

## Results

**Fig 2:**
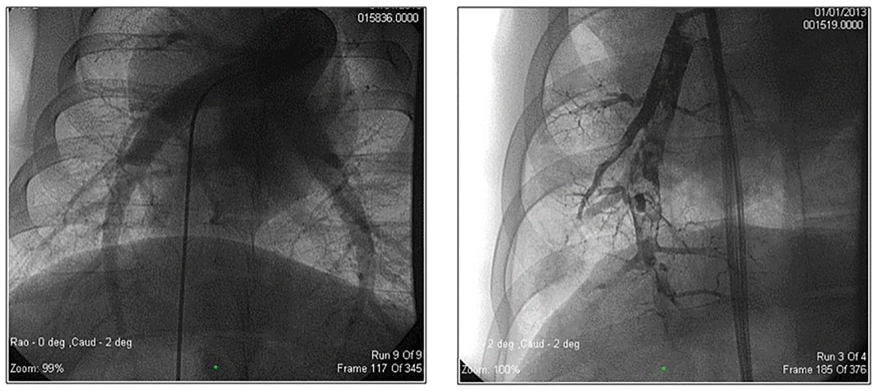
Infusion of dextran microspheres (100–300 μm) acutely obstructs pulmonary arteries. Representative still images from pulmonary artery angiographies obtained at (A) baseline or (B) immediately after embolization of microspheres.

All animals included in the study completed the embolization procedures per protocol and survived to the 8 week follow-up assessment timepoint. Pulmonary angiograms from before and immediately following the infusion of dextran microspheres (20 mg/kg) revealed that the procedure created acute subtotal luminal obstruction and diminished pulmonary vascular blood flow patterns (S1 and S2 videos). This finding, however, improved over time as the pulmonary angiograms from animals after repeated distal pulmonary embolization procedures demonstrated less luminal obstruction and better recovery of pulmonary blood flow compared to the initial post-embolization study (S3 video). Representative images from angiograms of the pulmonary arterial tree at baseline and immediately after embolization demonstrating acute occlusion of the arterioles are shown in Fig 2.

### Chronic increase in pulmonary artery pressure occurs in proximal + distal embolization model

**Fig 3:**
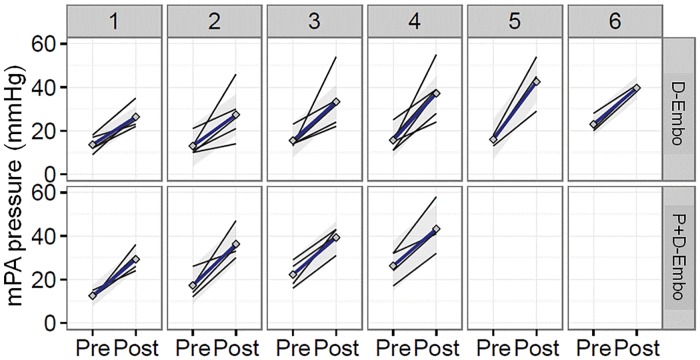
Acute changes in mPA pressure at the time of consecutive weekly embolization procedures. Hemodynamic assessments were made by right heart catheterization immediately before and after each weekly infusion of dextran microspheres. Animals in the D-Embo group (n = 6) underwent 6 embolization procedures (top) while pigs in the P+D-Embo group (n = 6) underwent 4 procedures (bottom). Changes for individual animals are plotted with the mean for the group shown as a blue line. mPA, mean pulmonary artery, P+D-Embo, proximal and distal embolization group; D-Embo, distal embolization group.

Although pulmonary artery pressures were elevated acutely ~2-fold after administration of the dextran beads in both models (Fig 3), pulmonary pressures returned to baseline between all of the subsequent embolization procedures in D-Embo animals (shown by the mPA pressure measured pre-embolization at each procedure). By contrast, there was a gradual increase in the mPA over time in P+D-Embo pigs. At the 8-week follow-up examination, indices of pulmonary arterial hypertension were present only in P+D-Embo animals (Table 1). Compared to sham controls and D-Embo pigs, mPA pressures were increased significantly in P+D-Embo pigs (14 ±1 vs. 16 ± 2 vs. 23 ± 4 mmHg, p<0.05) with normal PAOP observed in all groups. To examine the effect of the FiO_2_ on measured mPA pressures, the FiO_2_ was decreased to 21% for 10 min and mPA pressures were remeasured in sham and P+D-Embo animals. While there was no significant difference in mPA pressures at 21% compared to 40% FiO_2_ in sham pigs, there was an observed increase in mPA pressure at 21% in P+B-Embo animals (S1 Fig). The PVR index was also increased in P+D-Embo animals (3.9 ±1.6 WU*m^2^) compared to shams (2.0 ± 0.4 WU*m2) and D-Embo animals (2.5 ± 0.5 WU*m^2^). The pulmonary artery capacitance index, a measure of the pulsatility or compliance of the pulmonary artery, was decreased significantly in P+D-Embo pigs compared to D-Embo pigs (3.9 ±0.5 vs. 2.9 ±0.5, p<0.05) and inversely related to the PVR index. Thus, at 8 weeks, the combination embolization pigs all have evidence of chronic PH with increased vascular resistance and decreased compliance, which was not observed in animals that underwent distal embolization alone.

**Table 1:** Cardiopulmonary hemodynamics.

	Sham (n = 4)		D-Embo (n = 6)		P+D-Embo (n = 6)		P (ANOVA)
	Baseline	8 weeks	Baseline	8 weeks	Baseline	8 weeks	
**BW (kg)** ^**c**^ ^,^ ^**d**^ ^,^ ^**e**^	16±3	30±2	15±5	22±3	19±1	26±3	^a^P<0.05
**HR (bpm)** ^**d**^ ^,^ ^**e**^	69±10	65±14	85±10	72±13	62±4	71±15	^b^P<0.05
**Mean AoP (mmHg)**	88±15	96±27	75±9	74±7	78±12	82±19	NS
**PAOP (mmHg)**	5±1	5±2	5±3	6±2	3±1	9±3	NS
**mPAP (mmHg)** ^**d**^ ^,^ ^**e**^	17±5	14±1	13±4	16±2	14±3	23±4	^a^P<0.05
**TPG (mmHg)**	12±6	9±2	9±5	10±1	11±3	14±4	NS
**RA pressure (mmHg)**	3±1	2±1	3±1	4±2	2±1	2±2	NS
**Cardiac index**	3.5±1.0	4.4±0.4	4.1±1.3	4.3±0.8	3.7±0.4	3.7±0.6	NS
**SV index** ^**e**^	50±8	70±9	48±15	60±9	60±6	52±6	^a^P<0.05
PVR index (WU*m ^**2**^)	3.4±1.1	2.0±0.4	1.9±0.7	2.5±0.5	3.0±0.9	3.9±1.6	NS
**Capacitance index** ^**c**^ ^,^ ^**d**^ ^,^ ^**e**^	4.2±0.9	5.3±1.0	3.8±12	3.9±0.5	4.2±0.5	2.9±0.5	^a^P<0.05

BW, body weight; HR, heart rate; AoP, aortic pressure; PAOP, pulmonary artery occlusion pressure; mPAP, mean pulmonary artery pressure; TPG, transpulmonary gradient; RA, right atrium; SV, stroke volume; PVR, pulmonary vascular resistance; WU, Wood units.

^a^p<0.05: ANOVA for baseline comparisons.

^b^p<0.05: ANOVA for 8-week follow-up comparisons.

^c^p<0.05 post-hoc comparison: Sham vs. D-Embo^.^

^d^ p<0.05 post-hoc comparison: Sham vs. P+D-Embo.

^e^p<0.05 post-hoc comparison: D-Embo vs. P+D-Embo.

**Fig 4:**
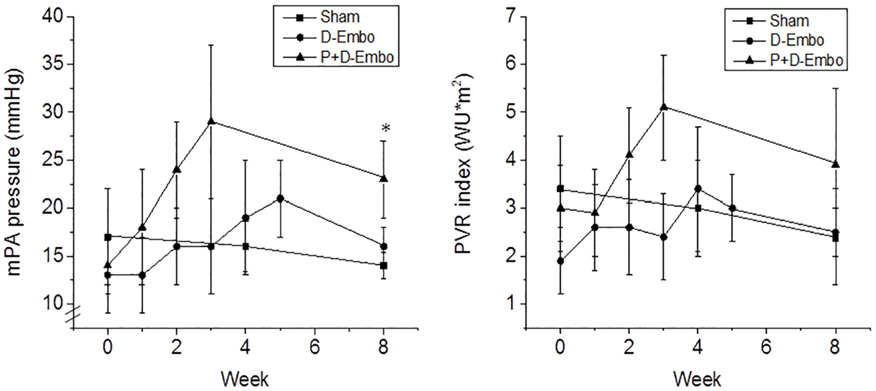
Temporal changes in mPA pressure and PVR index. The change over time in mPA pressure (*top*) and PVR index (*bottom*) measured at each embolization procedure and at the 8 week follow-up examination was evaluated in the P+D-Embo group (n = 6), D-Embo group (n = 6) and sham controls (n = 4). mPA, mean pulmonary artery; PVR, pulmonary vascular resistance; P+D-Embo, proximal and distal embolization group; D-Embo, distal embolization group. Data are reported as mean ± SD, *p<0.05 vs. sham, D-Embo by ANOVA.

The time course of the rise in mPA pressure and PVR index was also examined in both models (Fig 4). In D-Embo pigs, the peak mPA pressure measured was 21 ± 4 mmHg and was recorded at week 5, one week before the last embolization procedure; however, this increase in pressure was normalized at the 8 week follow-up examination and was similar to pressures recorded in sham controls. In contrast, in P+D-Embo pigs, mPA pressures increased by week 2, reached a maximum of 29 ± 8 mmHg (2.1-fold increase over baseline) by week 3, with some evidence of recovery to 23 ± 4 mmHg mmHg by week 8 (1.6-fold increase over baseline). The PVR index followed a similar temporal course with no difference observed between D-Embo animals and sham controls compared to their respective baseline measurements. In P+D-Embo pigs, the PVR index increased to 5.1 ± 1.1 WU*m^2^ at week 3 and by week 8 remained increased 82% over baseline, similar to the temporal trend observed for mPA pressures. These hemodynamic changes occurred without any observed differences between the groups with respect to the cardiac index or mean aortic pressure, both of which were preserved over time.

### Right ventricular dysfunction occurs in the proximal + distal embolization model

Consistent with the finding of PH in P+D-Embo pigs, echocardiographic examination revealed that there was also evidence of RV dysfunction (Table 2). Compared to shams and D-Embo pigs, there was a significant decrease in RVEF in P+D-Embo (70.0 ± 2.7 vs. 68.8 ± 7.0 vs. 58.8 ± 3.7, p<0.005) and a significant increase in the myocardial performance index (0.4 ± 0.1 vs. 0.4 ± 0.1 vs. 0.5 ± 0.1, p<0.006). Other indices of RV dysfunction were more pronounced in P+D-Embo compared to D-Embo animals, including abnormal longitudinal function as quantified by TAPSE and tissue Doppler imaging-derived peak myocardial velocity.

**Table 2:** Echocardiographic RV function and structural remodeling.

	Sham (n = 4)	D-Embo (n = 6)	P+D-Embo (n = 6)	P (ANOVA)
**EDV index**	101.7 ± 4.5	76.2 ± 14.1	104.4 ± 21.0	0.048
**ESV index**	30.5 ± 2.9	23.3 ± 4.1	43.3 ± 10.7^a^ ^,^ ^b^	0.006
**RVEF (%)**	70.0 ± 2.7	68.8 ± 7.0	58.8 ± 3.7^a^ ^,^ ^b^	0.005
**MPI**	0.4 ± 0.1	0.4 ± 0.1	0.5 ± 0.1^a^ ^,^ ^b^	0.006
**TDI peak S (cm/s)**	10.0 ± 0.9	8.6 ± 1.1	8.0 ± 1.0^a^	0.034
**TAPSE (mm)**	24.5 ± 2.4	21.6 ± 2.9	18.8 ± 2.4^a^	0.015
**RV weight (g/kg)**	1.14 ± 0.07	1.18 ± 0.09	1.42 ± 0.26	0.046
**RV/(LV+septum)**	0.40 ± 0.03	0.41 ± 0.02	0.47 ± 0.06	0.055

D-Embo, distal embolization model; P+D-Embo, proximal + distal embolization model; EDV, end-diastolic volume; ESV, end-systolic volume; RVEF, right ventricular ejection fraction; MPI, myocardial performance index; TDI peak S, tissue Doppler imaging peak systolic myocardial velocity; TAPSE, tricuspid annular plane systolic excursion; RV, right ventricle; LV, left ventricle.

^a^p<0.05 *post-hoc* comparison: Sham vs. P+D-Embo.

^b^p<0.05 *post-hoc* comparison: P+D-Embo vs. D-Embo.

**Fig 5:**
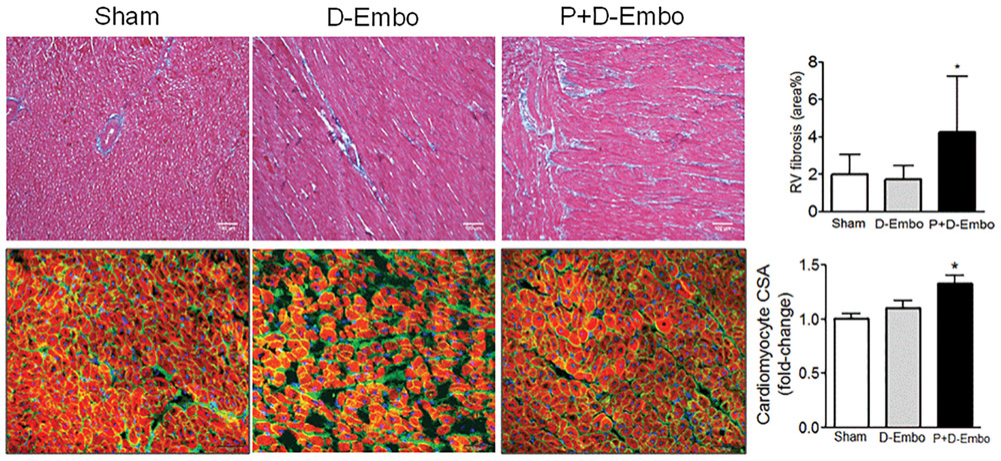
Right ventricular fibrosis and hypertrophy. Myocardial fibrosis was examined in RV sections from the P+D-Embo group (n = 6), D-Embo group (n = 6) and sham controls (n = 4) stained with Masson’s trichrome and quantified as % area fibrosis (*top*). Cardiomyocyte hypertrophy was evaluated by staining RV sections with wheat germ agglutinin and co-staining with phalloidin to assess cardiomyocyte cross-sectional area (*bottom*). Representative images are shown for each group. P+D-Embo, proximal and distal embolization group; D-Embo, distal embolization group; RV, right ventricular. *p<0.05 vs. sham, D-Embo by ANOVA.

There was also evidence of RV structural remodeling in P+D-Embo pigs. Compared to shams and D-Embo animals, the RV end-systolic volume index and end-diastolic index was increased significantly in P+D-Embo pigs. There was an increase in RV weight in P+D-Embo pigs (1.14 ± 0.07 vs. 1.18 ± 0.09 vs. 1.42 ± 0.26 gm/kg, p<0.05) and RV/LV + septum weight (0.40 ± 0.03 vs. 0.41 ± 0.02 vs. 0.47 ± 0.6 gm/kg, p<0.05) indicating that RV hypertrophic remodeling had occurred. To further characterize RV remodeling, myocardial interstitial fibrosis and cardiomyocyte hypertrophy were examined. Compared to sham and D-Embo animals, there was a significant increase in RV interstitial fibrosis in the P+D-Embo pigs (Fig 5). At a cellular level, there was evidence of RV cardiomyocyte remodeling with an increase in cardiomyocyte cross-sectional area observed only in in the P+D-Embo animals (Fig 5).

### Pulmonary vascular remodeling is present in the proximal + distal embolization model

**Fig 6:**
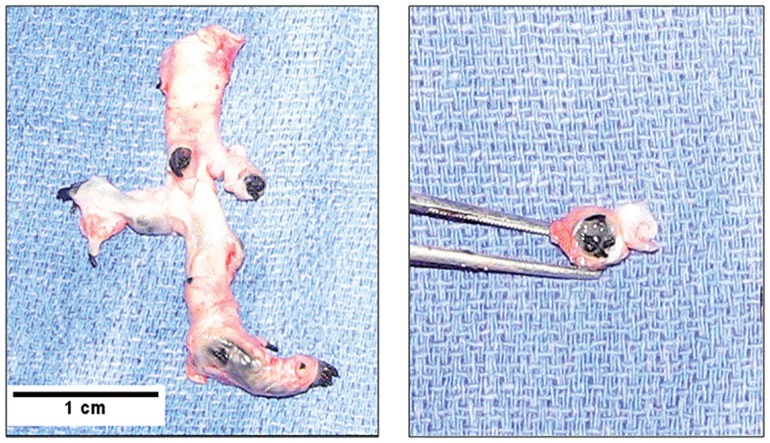
The combination proximal coiling and distal embolization protocol results in occlusion of pulmonary arteries. Explanted segments from the right inferior pulmonary artery showing silk coil and fibrin clusters obstructing the vessel lumen with dilation of the vessel. (*left*) Whole vessel segment explant; (*right*) cross-section through vessel showing silk suture and fibrin clusters occluding the lumen.

**Fig 7:**
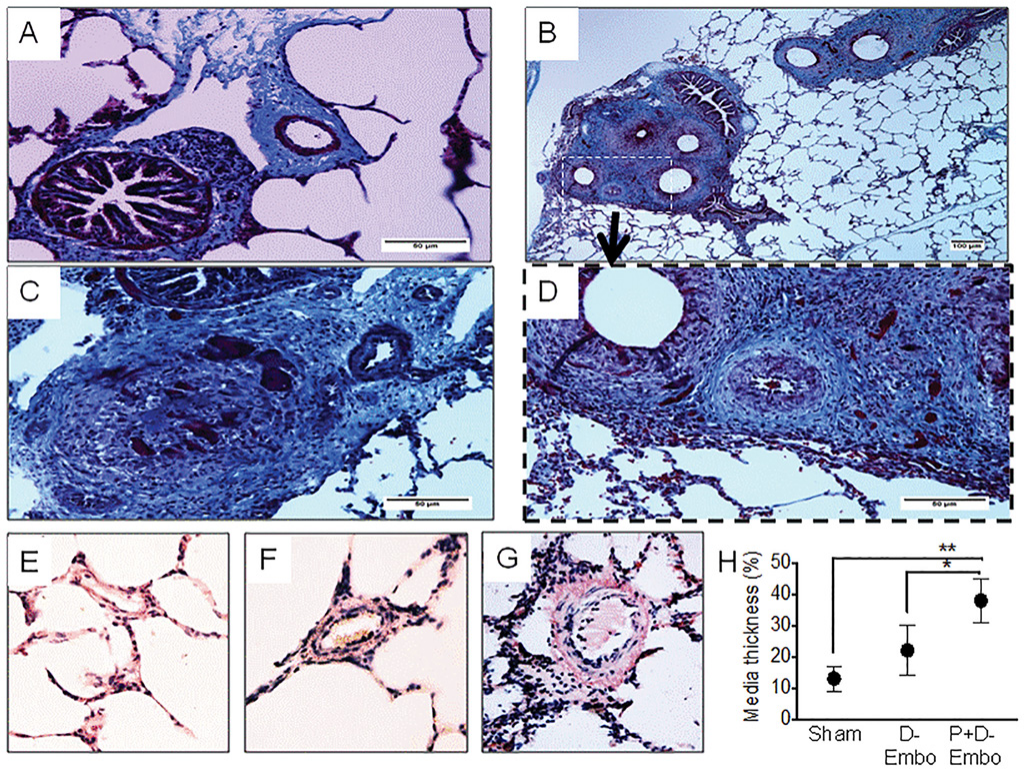
Pulmonary vascular remodeling. At 8 weeks, lungs were harvested, processed, and stained with Masson’s trichrome to examine pulmonary vascular remodeling. Representative images from A) sham and B-D) embolization models with similar patterns of remodeling, including luminal encroachment and hypertrophic intimal and medial remodeling, were observed for the D-Embo (B, D) and P+D-Embo pigs (C). In selected images (D), microspheres are present in the vessel lumen. There is also extensive perivascular collagen deposition. The distal pulmonary arteries were examined in the periphery of the lung in vessels that were not occluded by beads in sections stained with hematoxylin and eosin. Representative vessels from E) sham, F) D-Embo, and G) P+D-Embo animals are shown. H) Vessel media thickness as an indicator of pulmonary artery remodeling was assessed. P+D-Embo, proximal and distal embolization group; D-Embo, distal embolization group. Scale bars for A, B, C, and D are 60 μm, 100 μm, 60 μm, and 60 μm, respectively. *p<0.05 vs. D-Embo, **p<0.01 vs. sham by ANOVA.

Macroscopic examination of the lungs of P+D-Embo pigs showed clusters of silk and fibrin occluding the majority of branches of the pulmonary arteries (Fig 6). In both embolization models, dextran beads were present in the pulmonary artery lumen, which was narrowed significantly as a result of intimal and medial hypertrophic vascular remodeling. There was also evidence of extensive perivascular collagen deposition in the embolization models as compared with the sham controls. To determine the effect of proximal and distal embolization on distal pulmonary artery remodeling, we examined medial thickness in non-occluded distal pulmonary arteries in the lung periphery. This revealed that there was a significant increase in pulmonary arteriole medial thickening in theP+D-Embo animals as compared to the D-Embo and sham pigs (Fig 7).

## Discussion

Translation of scientific and therapeutic findings from PH studies using small animal models into the clinic has been limited, in part, by the lack of accessible large animal models that recapitulate the essential features of human disease. Investigators have attempted to create large animal models of PH utilizing recurrent intravascular embolization to simulate pulmonary arteriole obstruction with variable results. In many cases, PH did not develop, it occurred acutely but recovered, or studies were not carried out long-term to know if the model was durable over time. In the present study, we describe a novel swine model of chronic pulmonary vascular diseasecreated using a proximal coiling procedure combined with distal embolization (P+D-Embo). The key findings of the present study are: 1) a swine model of chronically elevated pulmonary artery pressures, RV dysfunction, and pulmonary vascular remodeling can be created by the combined embolization procedure; 2) the P+D-Embo protocol adds multiple lobar and segmental artery coiling to the standard D-Embo procedure and requires fewer intrapulmonary artery injections than the distal embolization alone protocol; 3) hemodynamic comparison between P+D-Embo and D-Embo animals reveals that the addition of proximal vessel coiling is an essential element to induce PH; 4) the P+D-Embo model also develops RV structural remodeling and RV dysfunction; and, 5) the procedure is safe with 0% mortality observed over the 8-week follow-up period.

There have been other studies that utilized recurrent embolization techniques to create a PH model in large animals (Table 3) [7,8,9,10,11,12,13,15,16]. These studies have a number of notable differences between them, including the embolic material (e.g., dextran beads, ceramic beads, air), number of procedures required to create the model (e.g., continuous/daily, weekly, biweekly), and the experimental conditions (e.g., mechanical ventilation, pO_2_, anesthesia) under which the cardiopulmonary hemodynamics were assessed. The importance of these variables is demonstrated by comparing our findings to those from a previous study that performed embolization in pigs using similar doses of microsphere. Our study reported a small, albeit more sustained, increase in mPA pressures and pulmonary hypertension than the other study[16], although pulmonary vascular resistance was similar. Differences between these studies could be explained by a different vascular response to the embolic injury in Yorkshire versus Large-White pigs that were used in each study as well as the different anesthesia protocols and oxygenation conditions at the time of hemodynamic evaluation, as shown in Table 3. The importance of oxygenation conditions is also underscored by our observation that the mPA pressure was lower in anesthetized animals ventilated with 40% FiO_2_ as compared to 21% FiO_2_. Another important variable is the time interval between the last embolization procedure and the final assessment (ranging from 1 hour to 6 months). This wide variability in experimental methodology is consistent with the fact that not all embolization protocols developed PH. It has been suggested that the models may have failed owing to pulmonary artery flow redistribution or the progressive recruitment or distension of existing capillaries [17]. It has also been estimated that increases in resting PA pressure don’t occur until >50% of the pulmonary circulation is obstructed [17]. Regarding the required amount of beads needed to cause significant levels of PH, Shelub et al [7] estimated that the number of microspheres injected greatly exceeded the number of vessels of similar or smaller diameter than the beads (~300 μm). They further speculated that: 1) flow would still occur despite the impacted beads due to vessel dilatation in response to the acute occlusion, and that 2) maldistribution of beads would spare a significant percentage of vessels that would eventually dilate and contribute to restoring pulmonary blood flow. With respect to the second explanation, we observed an acute increase in pulmonary pressures even at low doses of bead injection, suggesting that acute vasoconstriction may occur and affect the even distribution of beads across the pulmonary vasculature. Accordingly, a much higher dose or modification of the embolization material may be required to create at least moderate elevations in pulmonary pressures. In our study, we solved this issue by increasing the dose of dextran beads delivered (20 mg/kg) and prediluting the beads in saline to increase bead swelling and, thereby, bead diameter to facilitate obstruction. When considering the effect of recurrent pulmonary artery embolization on mPA pressure and PVR index, the greatest observed relative change (> 2-fold vs. baseline or controls) in these variables was achieved only in studies where hemodynamic assessments were made following a short (≤ 7 days) recovery period after the last embolization procedure. This finding appears to be independent of animal species, embolization material, or ventilation [8,9,15]. In the current study, we added a proximal embolization procedure together with repeat distal embolization to create the chronic PH model. The use of silk sutures for this purpose is advantageous owing to its ready availability and low cost as compared to commercial coils. The coiling or proximal embolization procedure was performed using silk suture material, which has been used previously to coil cerebral aneurysms[18]. When applied to the pulmonary arterial vasculature, repeated silk suture deployment using angiographic guidance results in progressive occlusion of the pulmonary artery branches. This, in turn, contributed to our finding of increased mPA pressures and PVR one month after the last embolization procedure.

**Table 3:** Comparison between studies utilizing embolization techniques to create large animal pulmonary hypertension models.

1st Author	Reference in text	Species	Embolic material	Embolizations(n)^a^	N	Oxygenation	Anesthesia during RHC	Recovery period	mPAP	Δ mPAP	Δ mPAP(%)	PVR	Δ PVR	Δ PVR (%)	RV/LV+S	Δ RV/LV+S	Δ RV/LV+S (%)	RV function assessment	Δ RV function (%)
D-Embo	Current study	Swine	Sephadex G50 (20 mg/kg)	6	6	40%	propofol	14 days	16 (2)	2	28	2.5 (0.45)^d^	0.5	25	0.41 (0.02)	0.01	3	Echocardiography	None
P+D-Embo	Current study	Swine	Sephadex G50 (20 mg/kg) + coil	4	6	40%	propofol	1 month	23 (4)	9	64	3.9 (1.6) ^d^	1.9	95	0.47 (0.06)	0.07	18	Echocardiography	-16 (RVEF)
Pohlmann *et al*.	11	Sheep	Sephadex G50 (0.375 g, ~6 mg/kg)	60	9	21% (SB)	none	1 day	35 (9)	18^b^	106	1.7 (0.66)	0.82	93	0.42 (0.03)	0.07	20	None	-
Kim *et al*.	10	Canine	ceramic beads (3 mm)	4	5	21%	halothane	6 months	17 (2)	5 ^b^	42	4.3 (0.83)	2.1	95	NR	NR	NR	None	-
Zhou *et al*.	15	Sheep	air (continuous)	8 weeks	4	21% (SB)	none	7 days	34 (5)	21 ^b^	162	4.5 (1.8)	3.7	462	0.36 (0.02)	0.09	33	None	-
Weimann *et al*.	9	Swine	Sephadex G50 (15 mg/kg)	3 (days 0, 7 and 49)	8	30%	ketamine	7 days	18 (3)	5	38	2.3 (0.8) ^d^	0.1	5	NR	NR	NR	None	-
Perckett *et al*.	8	Sheep	air (continuous)	12 days	5	21% (SB)	none	1 hour	23 (4)	9	64	7 (1.54)	3.36	92	0.38 (0.13)	0.06	19	None	-
Shelub *et al*.	7	Canine	Sephadex G50	Variable (16–30 weeks)	5	21% (SB)	none	>7 days	29 (4)	15 ^b^	107	8.3 (2.3)	6.3	315	0.54^c^	0.16	42	None	-
Garcia-Alvarez *et al*.	16	Swine	Sephadex G50	4(3–6)	9	21% (SB)	midazolam	2 month	27(3) ^e^	8	42	3.2(1.5) ^e^	0.3	10	NR	NR	NR	None	-
Mercier *et al*.	13	Swine	Histoacryl +Left PA ligation	5	5	NR	NR	7 days?	28.5 (3.8)	16.9	146	9.8(4.5)	5.1	107	NR	NR	NR	Echocardiography and PV loop	-50 (RVFAC)

Data expressed as mean (SD) unless stated otherwise. SB: spontaneous breathing.

^a^ Per protocol.

^b^Compared with baseline (vs. control group).

^c^Only reported in 2/5 cases.

^d^Indexed (BSA).

^e^Medians (IQR) reported.

NR = not reported. RHC, right heart catheterization; mPAP, mean pulmonary artery pressure; Δ mPAP, change in mean pulmonary artery pressure; PVR, pulmonary vascular resistance; Δ PVR, change in pulmonary vascular resistance; RV/(LV+S), right ventricular weight divided by left ventricular + septum weight (Fulton index); Δ RV/(LV+S), change in right ventricular weight divided by left ventricular + septum weight (Fulton index); RV, right ventricle.

Other non-surgical approaches such as the administration of monocrotaline or hypobaric hypoxia to create PH have been trialed in large animal models. In one study, monocrotaline (12 mg/kg) elevated mPA pressures (34.0 ± 1.7 mmHg) after 6 weeks and echocardiographic evaluation revealed a decreased pulmonary artery acceleration time and pulmonary artery valve ejection time indicating PH and RV dysfunction, respectively. The model was, however, incompletely characterized as PVR, systemic pressures, and other measures of RV function were not reported. These pigs also had an increase in RV weight consistent with RV hypertrophy and pathological examination of the lungs revealed hypertrophic vascular remodeling[19]. Although this model appears promising, it is susceptible to the same criticisms elicited by the monocrotaline rat model. In particular, this model doesn’t form obstructive pulmonary vascular lesions and PH has been attributed to sustained vasoconstriction [20,21,22]. Other investigators used chronic hypobaric hypoxia as a mechanism to induce PH; however, this methodology is species specific as it was found to induce PH in calves but not in sheep. Moreover, the inflammatory pulmonary vascular remodeling that occurs is reversible upon exposure to normoxia [3].

In the present study, we conducted a comprehensive analysis of RV structural and functional remodeling using advanced echocardiographic techniques. Using 3D-echocardiography, we found that the P+D-Embo model of PH developed significant RV remodeling with an increased RV end-systolic volume. Global RV performance (increased MPI) and longitudinal function (TAPSE) were also impaired significantly in the P+D Embo model. In prior studies using recurrent embolization models, RV function was not analyzed and reported systematically[23] (Table 3) despite the fact that it is a key contributor to the cardiopulmonary hemodynamics observed in PH and has prognostic implications. Among the studies that reported changes in relative RV mass as an indicator of RV hypertrophy, RV weight increased 18–42%, with greater increases in weight associated with higher mortality and likely indicative of worse RV function [7]. We also examined PA compliance as a measure of RV afterload and found lower PA compliance (increased RV afterload) in the P+D-Embo model [24,25]. This is consistent with the observation that pulmonary artery stiffness is an independent predictor of RV failure and has been shown to add prognostic information to hemodynamic markers of increased afterload such as PVR [24,25]. Moreover, histolopathological analysis of the pulmonary vasculature demonstrated hypertrophic vascular remodeling and increased collagen deposition, which likely contributes to the decrease in PA compliance and has been described previously [16]. Regarding the degree of RV dysfunction in relation to relatively mild hemodynamic chronic severity, our results indicate that decreased capacitance is an important contributor to early impairment of RV function and the development of myocardial hypertrophy and associated interstitial fibrosis,

### Limitations

There are several limitations associated with the findings of our study. Based on our initial goal of examining cardiopulmonary hemodynamics within a sub-acute and longer-term time frame, we examined these parameters 4 weeks after the final embolization procedure in the P+D-Embo model. It, therefore, remains unknown if the hemodynamic profile changes over a longer follow-up time period. It is also unknown if additional embolization procedures would have resulted in higher mPA pressures after 8 weeks although this is not likely based on the observed trend in pre- and post-embolization mPA pressures. Furthermore, we demonstrated that mPA pressures were actually higher in animals when ventilated with 21% FiO_2_ than with 40% FiO_2_ indicating that PH was present in our model although we did not measure mPA pressures in D-Embo animals under these conditions. Another limitation common to all embolization protocols is that the foreign material elicits an inflammatory response and possibly an immune response that may contribute to the observed pulmonary vascular histopathology. We also examined RV structure and function using advanced echocardiographic techniques but did not examine pressure-volume relationships using PV loops, which would have provided additional information about RV function and performance.

## Conclusions

In conclusion, we demonstrate a clinically relevant swine model of pulmonary vascular disease that is created by the combination of a percutaneous proximal coil embolization procedure with distal pulmonary vascular embolization. This requires fewer procedures than previously reported embolization protocols and results in elevated mPA pressure, PVR, pulmonary vascular remodeling, and RV dysfunction at one month after the final procedure. Taken together these findings indicate that the swine P+D-Embo model does recapitulate the cardiopulmonary hemodynamic profile of human PH with RV structural and functional remodeling. Owing to the relative ease with which this model is created as compared to surgical models, it is likely that the combination embolization swine model of PH will serve as a valuable model for future preclinical studies of PH.

## Supporting Information

S1 Fig The effect of oxygen on mPA pressures. To examine the effect of oxygen on mPA pressures, the FiO2 was decreased from 40% to 21% for 10 minutes and mPA pressures were remeasured in sham (n = 4) and P+D-Embo pigs (n = 6). P+D-Embo, proximal and distal embolization group *p<0.05 vs. sham, **p<0.001 vs. sham.
(TIF)

S1 Video Normal pulmonary artery angiogram. (MOV)

S2 Video Pulmonary artery angiogram immediately after embolization. (MOV)

S3 Video Pulmonary artery angiogram at the 8 week timepoint. (MOV)
