# Supplementary figures and images for: Combination Proximal Pulmonary Artery Coiling and Distal Embolization Induces Chronic Elevations in Pulmonary Artery Pressure in Swine
Source: PLoS One. 2015 Apr 29;10(4):e0124526. doi: 10.1371/journal.pone.0124526 (PMC4414513; doi:10.1371/journal.pone.0124526)

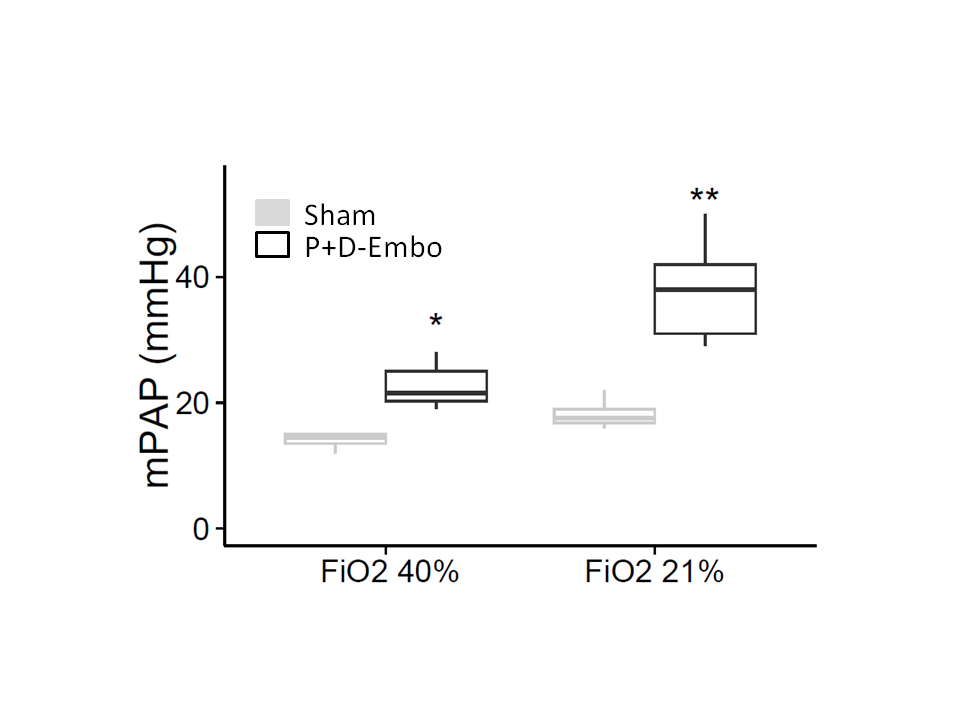

Supplement: S1 Fig — To examine the effect of oxygen on mPA pressures, the FiO2 was decreased from 40% to 21% for 10 minutes and mPA pressures were remeasured in sham (n = 4) and P+D-Embo pigs (n = 6). P+D-Embo, proximal and distal embolization group *p<0.05 vs. sham, **p<0.001 vs. sham. (TIF) [file pone.0124526.s001.TIF]
